# Pro-angiogenic and antibacterial copper containing nanoparticles in PLGA/amorphous calcium phosphate bone nanocomposites

**DOI:** 10.1016/j.heliyon.2024.e27267

**Published:** 2024-03-04

**Authors:** Lukas Näf, Iris Miescher, Lara Pfuderer, Tiziano A. Schweizer, David Brunner, Johannes Dürig, Olivier Gröninger, Julia Rieber, Gabriella Meier-Buergisser, Katharina Spanaus, Maurizio Calcagni, Philipp P. Bosshard, Yvonne Achermann, Wendelin J. Stark, Johanna Buschmann

**Affiliations:** a Department of Plastic Surgery and Hand Surgery, University Hospital of Zürich, Rämistrasse 100, 8091, Zürich, Switzerland; b Institute for Chemical and Bioengineering, Department of Chemistry and Applied Biosciences, ETH Zurich, CH-8093, Zurich, Switzerland; c Department of Dermatology, University Hospital Zurich, University of Zurich, Rämistrasse 100, 8091, Zurich, Switzerland; d Clinical Chemistry, University Hospital Zurich, 8001, Zurich, Switzerland

**Keywords:** Nanoparticles, Copper-doped tricalcium phosphate, Copper oxide, CAM assay, qPCR, VEGF, Ang1

## Abstract

Large bone defects after trauma demand for adequate bone substitutes. Bone void fillers should be antibacterial and pro-angiogenic. One viable option is the use of composite materials like the combination of PLGA and amorphous calcium phosphate (aCaP).

Copper stimulates angiogenesis and has antibacterial qualities. Either copper oxide (CuO) nanoparticles (NPs) were therefore added to PLGA/aCaP/CuO in different concentrations (1, 5 and 10 w/w %) or copper-doped tricalcium phosphate NPs (TCP with 2% of copper) were electrospun into PLGA/CuTCP nanocomposites.

Bi-layered nanocomposites of PLGA/aCaP with different copper NPs (CuO or TCP) and a second layer of pristine PLGA were fabricated. Two clinical bacterial isolates (*Staphylococcus aureus* and *Staphylococcus epidermidis*) were used to assess antibacterial properties of the copper-containing materials. For angiogenesis, the chorioallantoic membrane (CAM) assay of the chicken embryo was performed.

The higher the CuO content, the higher were the antibacterial properties, with 10 % CuO reducing bacterial adhesion most effectively. Vessel and cell densities were highest in the 5 % CuO containing scaffolds, while tissue integration was more pronounced at lower CuO content. The PLGA/aCaP/CuO (1 % CuO) behaved similar like PLGA/CuTCP in all angiogenic and antibacterial readouts, based on the same copper fraction.

We conclude that CuO NPs or CuTCP NPs are useful components to increase angiogenic properties of nanocomposites and at the same time exhibiting antibacterial characteristics.

## Graphical abstract

**Figure:**
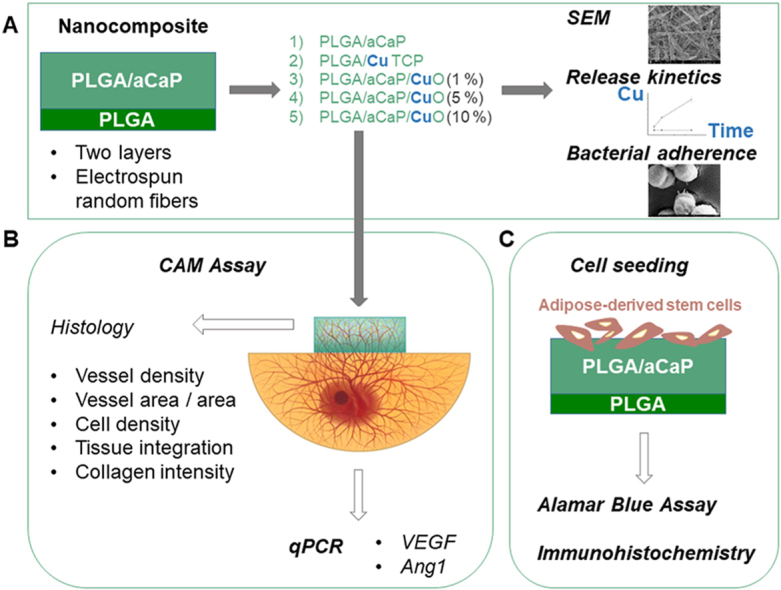

## Abbreviations

aCaP: amorphous calcium phosphate
Ang1: angiopoietin 1
ANOVA: analysis of variance
ASCs: adipose derived stem cells
α-SMA: alpha smooth muscle actin
BCI: bacterial clinical isolate
BTE: bone tissue engineering
CAM: chorioallantoic membrane
CuO: copper oxide
CuTCP: copper-doped (2 %) tricalcium phosphate
eNOS: endothelial nitric oxide synthase
EtOH: ethanol
FGF-1: fibroblast growth factor-1
HIF-1α: hypoxia inducible factor-1α
ICP-MS: inductively coupled plasma mass spectroscopy
NPs: nanoparticles
PLGA: poly lactic co glycolic acid
qPCR: quantitative polymerase chain reaction
ROS: reactive oxygen species
S. aureus: Staphylococcus aureus
S. epidermidis: Staphylococcus epidermidis
SEM: scanning electron microscopy
TCP: tricalcium phosphate
VEGF: vascular endothelial growth factor
w/w: weight per weight
XRD: X-ray diffraction
ZnO: zinc oxide

## 1 Introduction

Artificial bone substitutes in orthopedics demand for several characteristics to achieve a fast and proper function in the body. Besides adequate mechanics, osteoinduction, osteoconduction and osteogenic properties [1], an ideal bone substitute should support angiogenesis [2]. As *de novo* formation of blood vessels, angiogenesis is a prerequisite for a regenerative integration of bone substitutes; to provide a fast delivery of oxygen and nutrients to the whole substitute - including the core, where particularly in implants for critical size bone defects necrosis may occur, which is one of the big challenges in bone tissue engineering (BTE).

Many material-related approaches exist to fabricate bone substitutes, including ceramics [3], polymers [4] and composite materials [5]. Besides 3D printing [6], electrospinning techniques are considered in BTE [7]. In our research group, we have tested a nanocomposite developed at ETH Zurich under Prof. Wendelin J. Stark [8]. The material is PLGA/aCaP consisting of electrospun PLGA fibers with incorporated nanoparticles (NPs) of amorphous calcium phosphate (aCaP), which showed promising properties under *in vitro* conditions [9,10] and *in vivo* [11,12]. To further modify this nanocomposite for improvement of angiogenic characteristics, we incorporated copper oxide NPs (CuO NPs) to result in a PLGA/aCaP/CuO nanocomposite. This material showed enhanced angiogenesis in a murine model for chest wall replacement when compared to PLGA/aCaP [13]. We had incorporated the CuO NPs in a 5 w/w %. However, we did not investigate dose-response with different CuO NPs concentrations so far.

Besides being pro-angiogenic, CuO NPs exhibit antibacterial properties [14]. Contamination with common colonizing bacteria originating from the patient's own skin, such as *staphylococci* [15,16], can occur and might result in implant-associated osteomyelitis. Bone infection is devastating, but many artificial bone substitutes with excellent biocompatibility and mechanical strength do not have antibacterial properties [17]. Metal oxide NPs may exhibit both, pro-angiogenic and antibacterial characteristics, respectively [18]. Among them, for example ZnO in a PCL/Ta/ZnO artificial periosteum has been reported to have brilliant pro-angiogenic and bactericidal properties [19]. Also Cu-based biomaterials have been reported to be highly effective [20,21]. Therefore, a dose-response investigation for CuO NPs in PLGA/aCaP/CuO is relevant for an optimized function of such artificial bone substitutes, because too high concentrations may be not only detrimental to bacteria, but also to cells and in turn impede bone healing.

We also used flame spray pyrolysis to synthesize novel tricalcium phosphate (TCP) NPs doped with 2 % of copper (CuTCP), where copper is located within the CuTCP NPs like in a Trojan horse. These CuTCP NPs were synthesized as an alternative to CuO NPs. As Cu is “buried” within CuTCP, copper release kinetics from CuTCP NPs were assumed to be different than from CuO NPs. Furthermore, the release kinetics were supposed to be influenced by the pH of the microenvironment [22], similar to earlier reported silver release from AgTCP NPs [23].

**Scheme 1:**
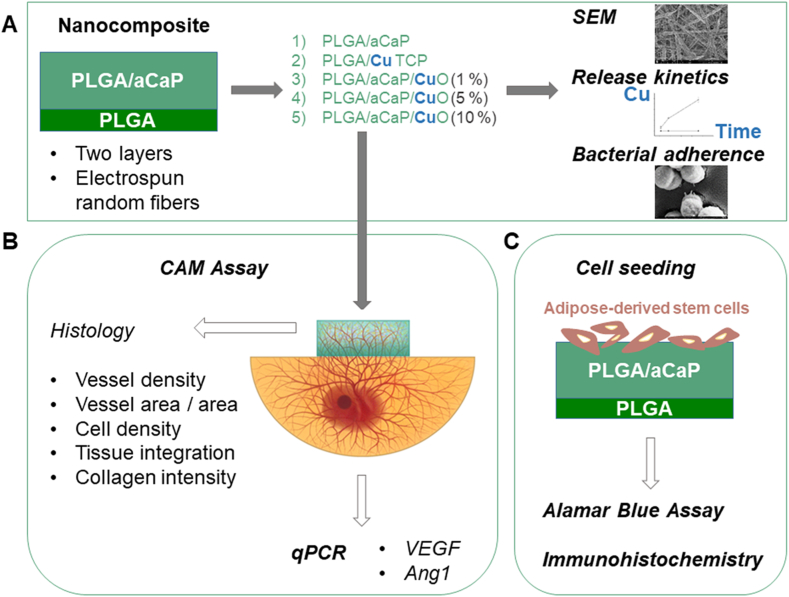
Overview of nanocomposite production and experiments. Fabrication of five bi-layered electrospun random fiber meshes with a thin layer of pristine PLGA (dark green) and a thick layer of a nanocomposite with the different compositions (1–5); and characterization by scanning electron microscopy (SEM), assessment of copper release kinetics over time and bacterial adherence (**A**), CAM assay with histological assessment and quantitative real-time PCR (qPCR) for *VEGF* and *Ang1* gene expression (**B**), and seeding of adipose-derived stem cells on nanocomposites to measure proliferation by Alamar blue assay and immunohistochemical labeling after fixation (**C**).

After X-ray diffraction (XRD) characterization of the three kinds of NPs used here (aCaP, CuO and CuTCP NPs), the electrospun nanocomposites were imaged by scanning electron microscopy (SEM). Growth of *Staphylococcus aureus* and *Staphylococcus epidermidis* on these materials was tested quantitatively as well as qualitatively by SEM. The chorioallantoic membrane (CAM) assay [24] was used to assess angiogenic parameters like vessel density after a 1-week incubation of the nanocomposites on top of the CAM *in ovo*. It was hypothesized that angiogenic response is increased with increasing amount of CuO in PLGA/aCaP/CuO. Gene expression of *VEGF* and *Ang1* in the CAM tissue underneath the corresponding scaffolds has been assessed as well. Finally, rabbit adipose-derived stem cells were seeded onto these nanocomposites, and the proliferation was measured with Alamar Blue assay. Immunohistochemical labeling for collagen I, fibronectin, and α-SMA in the extracellular matrix expressed by these cells complete our study (Scheme 1).

The objectives were: (i) to characterize and compare the aCaP, CuO and novel CuTCP NPs and the different electrospun nanocomposites, (ii) to determine the best CuO NP concentration in PLGA/aCaP/CuO nanocomposite with respect to both, pro-angiogenic and antibacterial properties, and (iii) to compare PLGA/CuTCP with PLGA/aCaP/CuO (1 w/w %) as they exhibit the same copper fraction, but in two different formulations, either as CuO NPs or CuTCP NPs, representing a novel kind of NPs.

## 2 Materials and methods

### 2.1 Nanoparticle preparation

Amorphous tricalcium phosphate NPs with 2 % of copper (CuTCP) or without copper (aCaP with Ca:P = 1.5 [25]) were prepared by flame spray synthesis [26] and a size of ∼22 nm. For aCaP NPs, calcium-2-ethyl-hexanoic salt (synthesized by calcium hydroxide from Riedel de Haen, Ph. Eur. and ethylhexanoic acid from Sigma-Aldrich, St. Louis, USA) and tributyl phosphate (98% Sigma-Aldrich) were used. The CuO NPs were received as Copper nanopowder from Sigma Aldrich (40–60 nm (SAXS)). NPs were characterized with X-ray diffraction (XRD).

### 2.2 Electrospun nanocomposites

PLGA with 85 % lactic acid and 15 % glycolic acid was received from Boehringer Ingelheim am Rhein, Germany. The different components were combined, as reported previously for PLGA/aCaP [11]. Hybrid nanocomposites were fabricated by electrospinning of two layers; with a ∼500 μm thick layer of PLGA/aCaP/CuO (with different w/w of CuO NPs) or PLGA/CuTCP, and an approximately 30 μm thick layer of pristine PLGA for all nanocomposites. The hybrid nanocomposites were investigated with scanning electron microscopy (SEM, FEI, Nova NanoSEM 450).

Disks with a diameter of 1 cm and a height of ∼530 μm were fabricated for the five types of nanocomposites (Table 1). They were immersed into a 1 % solution of penicillin-streptomycin (Sigma #P4333-100 ML) in a phosphate buffer solution (Dulbecco's Phosphate Buffered Saline, Sigma #D8537-1L) for 24 h before placing them on the CAM. For the microbiological part of the study, the nanocomposites were sterilized with UV light for half an hour on both sides.

**Table 1:** Composition of the bi-layered electrospun random fiber meshes. The percentage (%) is indicated on a weight/weight (w/w) basis.

Nanocomposite	First layer	2nd layer
PLGA/aCaP	60 % PLGA +40 % aCaP	PLGA
PLGA/CuTCP	60 % PLGA +40 % Cu(2%)TCP	PLGA
PLGA/aCaP/CuO (1%)	60 % PGLA + 39 % aCaP + 1 % CuO	PLGA
PLGA/aCaP/CuO (5%)	60 % PGLA + 35 % aCaP + 5 % CuO	PLGA
PLGA/aCaP/CuO (10%)	60 % PGLA + 30 % aCaP + 10 % CuO	PLGA

### 2.3 In vitro copper release kinetics

In order to assess the Cu release kinetics from PLGA/aCaP/CuO with different amounts of CuO NPs, disks were incubated in 1 mL of Aqua B. Braun water (B. Braun company, Melsungen AG, Switzerland) at 37 °C (n = 2). For the PLGA/CuTCP nanocomposites, release kinetics at different pH values were assessed; pH 5.5 (MES buffer), pH 7.0 (PIPES buffer), and pH 7.4 (PIPES buffer), respectively (n = 2). The nanocomposites were submersed in micro test tubes containing 1 mL of the respective buffer solution and incubated at 37 °C. Samples of 1 mL volume were taken on day 1, day 8, and day 36 after incubation and replaced with new buffer solution – or in case of the PLGA/aCaP/CuO nanocomposites with 1 mL Aqua B. Braun water. All samples were stored in Eppendorf tubes at 4 °C until the copper concentrations were determined using an Agilent 8900 ICP mass spectrometer (Agilent Technologies, Switzerland). For the analysis samples were diluted with 1 % HNO_3_ and analyzed using He gas mode with Rhodium as an internal standard and analyzed in triplicates. Internal controls (ClinCheck, Recipe, Munich Germany) were analyzed with each batch of samples to ensure the quality of the data produced. Cumulative release kinetics were calculated by add-on to concentrations at earlier time points.

### 2.4 Bacterial adherence

To analyze bacterial adhesion and biofilm formation on the nanocomposites, small disks (diameter 5 mm) of the nanocomposites were prepared with a sterile biopsy puncher and placed in a Petri dish. The disks were sterilized with UV light for half an hour on each side and placed in 96-well plates. *Staphylococcus aureus* (BCI175) and *Staphylococcus epidermidis* (BCI195) from patients suffering with peri-prosthetic joint infection were used [27] To obtain fresh cultures, bacteria were grown from frozen cultures on Brain Heart Infusion (BHI, Becton Dickinson, Heidelberg, Germany) agar plates for 24 h at 37 °C. Liquid cultures were prepared by picking multiple single colonies from agar plates and inoculating 5 mL BHI broth at 37 °C with 220 rounds per minute (rpm) agitation overnight. The next day, bacterial cultures were centrifuged at 1800 g for 5 min and the pellets resuspended in phosphate-buffered saline (PBS) and afterwards diluted in Roswell Park Memorial Institute (RPMI) 1640 (Gibco™, 11875093) with 10 % fetal bovine serum to obtain a final density of approximately 10^6^ colony forming units of bacteria per mL (CFUs/mL). The nanocomposites were infected with 200 μL of the bacterial suspension (corresponding to 2 × 10^5^ CFUs), and incubated at 37 °C and 5 % CO_2_. After 24 h the medium was removed from the wells and the nanocomposites were relocated to a new 96-well plate followed by washing them 3 times with 200 μL PBS before adding 200 μL of PBS. In a further step, the nanocomposites were sonicated for 1 min at 40 kHz. Afterwards, the bacterial suspensions were diluted 10-fold, drop-plated (20 μL drops) on agar plates and incubated for 24 h at 37 °C before counting and determination of the CFUs/mL. In total three biological replicates of each strain were evaluated in technical duplicates. For SEM, nanocomposites were inoculated with *S. aureus* for 24h, after which the medium was removed, the nanocomposites washed 3 × with 200 μl PBS and finally fixed with 1% GA and 4% PFA and stored at 4 °C until further sample preparation.

### 2.5 Isolation of rabbit adipose-derived stem cells (ASCs)

Rabbit ASCs were isolated from fat tissue of New Zealand White rabbits (Approval by the veterinary office of Canton Zurich, reference number ZH 080/2021; 33530). Briefly, fat tissue was washed with Phosphate Buffered Saline (PBS) (Sigma-Aldrich, Merck, Buchs, Switzerland) supplemented with 100 U/mL penicillin (P), 100 μg/mL streptomycin (S), 1% GlutaMAX (G) (ThermoFisher Scientific, Basel, Switzerland) and 2.5 μg/mL amphotericin B (A) (Pan Biotech, Aidenbach, Germany). Tissue samples were minced in a sterile tissue culture plate using two scalpels and transferred into a sterile 50 mL Falcon tube. Fat tissue was digested with collagenase Type A (Roche, Basel, Switzerland) at a final concentration of 1 mg/ml prepared in 1–20 mL DMEM-HG with P/S/A/G, shaking for up to 1 h at 37 °C in a water bath. Collagenase activity was neutralized by adding an equal volume of DMEM-HG supplemented with P/S/A/G and 10% heat inactivated Fetal Bovine Serum (FBS) (Biowest, Nuaillé, France). After disintegrating the samples by pipetting up and down, they were transferred through a 70 μm cell strainer (Miltenyi Biotec Swiss AG, Solothurn, Switzerland) into a fresh 50 mL tube and centrifuged at 400×*g* for 10 min. Collagenase solution was aspirated and pellet was resuspended in DMEM-HG containing P/S/A/G and 10% FBS. Cell suspension was transferred into sterile petri dishes and incubated at 37 °C, 5% CO2. Non-adherent cells were removed after 24 h and medium was replaced every 3–4 days.

### 2.6 Cell seeding and proliferation assay on nanocomposites

PLGA/aCAP and PLGA/aCAP/CuO (5%) nanocomposites were sterilized in PBS containing 200 μg/mL gentamycine (Biowest, Teco Medical, Sissach, Switzerland) and 2.5 μg/mL amphotericine B (Biowest, Teco Medical, Sissach, Switzerland) for 24 h and dried in the sterile bench. Half of the nanocomposites were shortly wetted in 50 % ethanol afterwards, rinsed three times with water and washed once with cell culture medium DMEM-HG (ThermoFisher Scientific, Basel, Switzerland) containing 1% P/S/G (ThermoFisher Scientific, Basel, Switzerland) and 10% FBS (Biowest, Nuaillé, France). Circles of 5 mm diameter were punched out of the nanocomposite material and placed in a 12-well plate (Sigma-Aldrich, Merck, Buchs, Switzerland). ASCs from two donors (P 7, 5 × 10^5^ cells in 10 μL medium) were seeded on top and allowed to settle at 37 °C and 5 % CO_2_ for about 3 h before adding 1 mL cell culture media to each well. The culture media was exchanged every third day and the experiment was carried out in technical duplicates. Cell proliferation was determined on day 3, 7 and 14 by Alamar Blue™ cell viability assay (ThermoFisher Scientific, Basel, Switzerland). The nanocomposites were transferred into a 96-well plate (Techno Plastic Products AG, Trasadingen, Switzerland) and incubated with 100 μL 1:10 diluted Alamar Blue™ solution for 4 h before excitation wavelength of 530 nm and emission wavelength of 590 nm was measured using a Cytation 5 imaging reader (BioTEk, Agilent Technologies AG, Basel, Switzerland). After measurement, nanocomposites were washed once with medium before adding 1 mL medium for further cultivation. Empty wells were filled with PBS to prevent dehydration. After measurement on day 14 nanocomposites were fixed in 4 % paraformaldehyde (PFA) for further immunocytostaining.

### 2.7 CAM assay

The CAM assay was performed as previously described [28]. Briefly, on incubation day (ID) 3.5 of the eggs, the eggshell of fertilized Lohmann white LSL chicken eggs (Animalco AG Geflügelzucht, Switzerland) was windowed after removal of ∼2 mL of egg white. No IACUC approval is required until embryonic day 14 according to Swiss animal care guidelines (TSchV, Art. 112). The fertilized chicken eggs were incubated at 37 °C and 65 % relative humidity. The egg window was covered with the bottom lid of a sterile Petri dish and the eggs were incubated again. Nanocomposite grafting was performed on ID 8. The nanocomposites were placed within a silicone ring of 1 cm in diameter on the CAM, with the copper-containing layer facing the embryo (control PLGA/aCaP layer). The eggs were again incubated until ID 14.

### 2.8 Histology

Fixation of the nanocomposites was conducted overnight using formaldehyde (4%) on ID 14. The nanocomposites and silicone rings were excised and cut in half before they were embedded in paraffin. The 3 μm thick tissue sections were stained with hematoxylin and eosin (H&E) for the histological evaluation of the vessel density, vessel area, cell density and tissue integration. The semi-quantitative analysis of the collagen intensity was accomplished using Masson Goldner Trichrome staining (scores low, medium and high collagen intensity). The histological sections were scanned at the Center for Microscopy and Image Analysis (ZMB), University of Zurich. The collection of the data for the vessel density, area of vessels per area, cell number per area, and collagen intensity was performed in 100 μm × 100 μm squares in three regions; interface, middle and surface, which were each defined as one third of the distance from the interface of the CAM to the top of the nanocomposite. Evaluation of the parameters was done in five squares per region, which made up for a total of 15 squares per slide. The tissue integration was measured in five different areas in the nanocomposite on each slide. The number of eggs included in histological analysis was n = 19 for PLGA/aCaP; n = 6 for PLGA/CuTCP; n = 4 for PLGA/aCaP/CuO (1 %); n = 9 for PLGA/aCaP/CuO (5 %) and n = 1 for PLGA/aCaP/CuO (10 %). The analysis was conducted using a Leica DM 6000 microscope with a Leica DMC 2900 camera.

### 2.9 Immunocytochemistry

ASCs seeded nanocomposites were embedded in paraffin according to commonly established protocols and cut into 3 μm thick slices before de-paraffinization with xylene and subsequent rehydration. Sections underwent an antigen retrieval step in 10 mM citrate buffer (pH 6.0) (Sigma-Aldrich, Merck, Buchs, Switzerland) with 0.05% Tween-20 (Sigma-Aldrich, Merck, Buchs, Switzerland) for 20 min at 95 °C. After cooling, samples were washed 3 times with 1× Tris-buffered saline (TBS). Samples for α-smooth muscle actin (α-SMA) staining were permeabilized with 0.5% Triton X-100 (Sigma-Aldrich, Merck, Buchs, Switzerland) in 1× TBS for 10 min and washed 3 times with 1× TBS afterwards. Sections were quenched with 3 % H_2_O_2_ (Sigma-Aldrich, Merck, Buchs, Switzerland) in deionized water for 10 min at room temperature and washed subsequently 3 times with 1× TBS before samples were blocked in 1× TBS supplemented with 5% donkey serum and 1% Bovine Serum Albumin (BSA) (Sigma-Aldrich, Merck, Buchs, Switzerland) for 1 h at room temperature. Afterwards, sections were incubated at room temperature for 30 min with mouse anti collagen I antibody (Abcam, Lucerna-Chem AG, Luzern, Switzerland; 1:200 dilution), mouse anti fibronectin antibody (Sigma-Aldrich, Merck, Buchs, Switzerland; 1:200 dilution) or mouse anti α-SMA antibody (Sigma-Aldrich, Merck, Buchs, Switzerland; 1:500 dilution) diluted in 1× TBS containing 3% BSA. Slides were washed with tap water and 3 times 2 min with 1× TBS before primary antibody detection was carried out with a ZytoChem Plus (HRP) Anti-Mouse (DAB) Kit according to the manufacturer's protocol (Zytomed Systems, Berlin, Germany). Hematoxylin staining (DAKO, Agilent Technologies, Basel, Switzerland) was performed for 10 min. The slides were washed in tap water, dehydrated with ethanol and mounted with Pertex. Slides were imaged for qualitative analysis at 100 × magnification using a confocal microscope (Leica AF 6000B).

### 2.10 qPCR

The nanocomposites on the CAM were also used for qPCR. For that purpose, the nanocomposites were manually shredded into smaller pieces using a pipette tip. Using the RNeasy Plus Mini Kit (QIAGEN®, Stockach, Germany) RNA was isolated according to the protocol. Concentration and purity of RNA was analyzed by NanoDrop Spectrophotometer (ThermoFisher Scientific, Waltham, USA). RNA was reverse transcribed into cDNA analog to the protocol using oligo dT-primers (Invitrogen, Carlsbad, CA, USA) and SuperScript III reverse transcriptase. The actual qPCR was done by the QuantStudio5 (Applied Biosystems) machine using the fast SYBR® Green Master Mix (Applied Biosystems, Waltham, USA) and a specific amplification program of the QuantStudio Design Software v.1.4.3. The raw data was transcribed to Microsoft Excel program and the fold expression change was calculated using the 2^−ΔΔCT^ method [29]. The expression of two pro-angiogenic target genes, *VEGF* and *Ang1*, was analyzed while *β-actin* served as reference gene.

### 2.11 Statistics

For the statistical analysis, IBM SPSS software was used (Version 26). After checking normal distribution of data (Shapiro Wilk) and variance homogeneity (Levene), either parametric 1-way ANOVA or non-parametric Kruskal-Wallis test was utilized to compare more than two groups. For a 2-group only comparison, either unpaired *t*-test (parametric) or Mann Whitney *U* test (non parametric) was applied. Data are presented as mean ± standard deviation. Significance was considered if p < 0.05 (*), p < 0.01 (**) or p < 0.001 (***).

## 3 Results

### 3.1 Characterization of NPs and nanocomposites

**Fig. 1:**
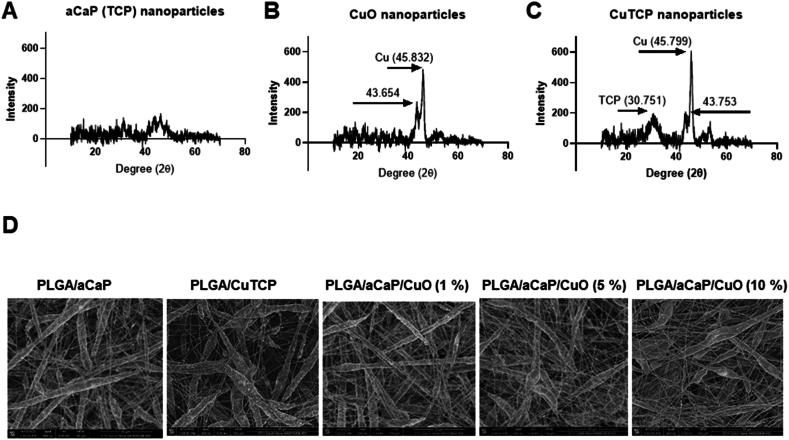
XRD spectra of NPs used: amorphous calcium phosphate (aCaP) (**A**), copper oxide (CuO) (**B**), and copper-doped amorphous calcium phosphate (CuTCP) (**C**). SEM images of PLGA/aCaP, PLGA/CuTCP and PLGA/aCaP/CuO nanocomposites with varying CuO concentrations (**D**).

Three types of NPs were used for the fabrication of the nanocomposites. These NPs were characterized by XRD (Fig. 1 A, B and C).

While aCaP NPs showed a diffuse signal in the XRD spectrum at approximately 30° and 43° (Fig. 1**A**), CuO NPs exhibited distinct peaks at 43.654° and 45.832° (Fig. 1B). When TCP was doped with 2 w/w % of copper, the resulting CuTCP NPs showed both, the typical signals for copper and for the aCaP NPs, respectively (Fig. 1C).

Electrospun bi-layered fiber meshes, with a first layer composed of either (i) PLGA with dispersed aCaP NPs, (ii) PLGA with dispersed CuTCP NPs, and (iii) PLGA with an incorporated mixture of dispersed aCaP NPs and CuO NPs in different ratios; and all with a second thin layer of pristine PLGA to stabilize the material (Table 1), were characterized by SEM (Fig. 1D). The PLGA/aCaP/CuO and PLGA/CuTCP layer were approximately 500 μm thick and the pure PLGA layer was about 30 μm thick [12].

### 3.2 Copper release kinetics

**Fig. 2:**
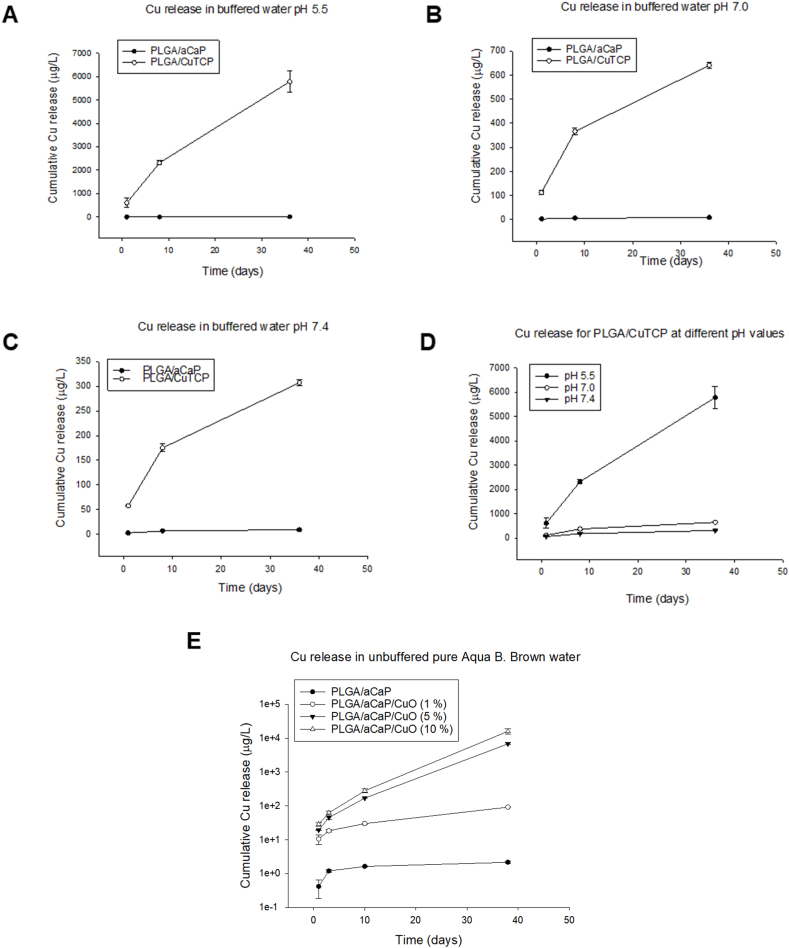
Cu release kinetics of PLGA/CuTCP are influenced by pH value (**A-D**). For PLGA/aCaP/CuO nanocomposites, Cu release depends on the incorporated amount of CuO NPs (**E**).

The copper release kinetics of the different nanocomposites were assessed (Fig. 2). Low pH values favored Cu release from PLGA/CuTCP meshes (Fig. 2 A, B, C and **D**). For the PLGA/aCaP/CuO nanocomposites, the higher the incorporated concentration of CuO NPs, the higher the released Cu concentration at each respective time point (Fig. 2 E).

### 3.3 Bacterial adherence

**Fig. 3:**
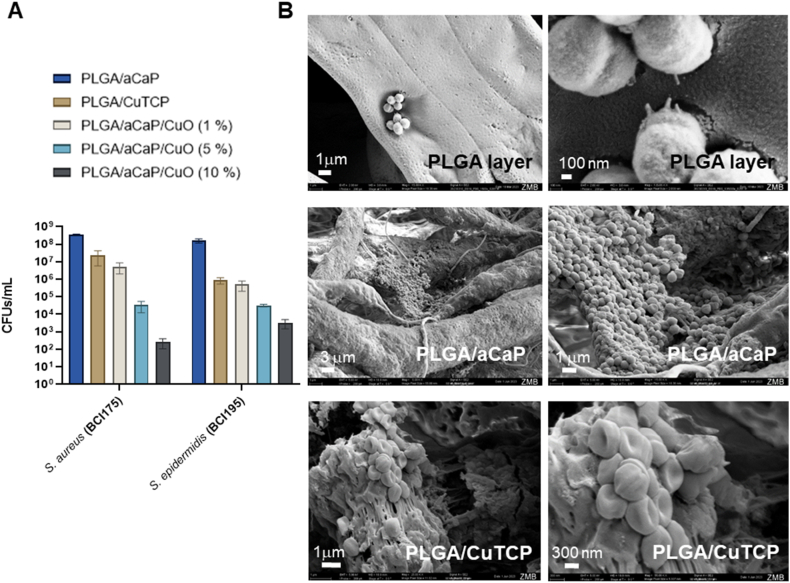
Two clinical bacterial isolates were used to assess the antibacterial activity of copper within the different (n = 3). The higher the copper content, the lower the bacterial adherence (**A**). SEM imaging of *S. aureus* (BCI175) on pure PLGA layer (top row), on PLGA/aCaP layer (middle row), and on PLGA/CuTCP layer (bottom row) (**B**). *Key*: *S. aureus* = *Staphylococcus aureus*; *S. epidermidis* = *Staphylococcus epidermidis*; BCI = Bacterial Clinical Isolate. Statistical analysis by 1-way ANOVA revealed that for both strains all materials were significantly different from each other, except for the pair of PLGA/aCaP/CuO (1 %) and PLGA/CuTCP.

For two clinical bacterial isolates, *S.aureus* (BCI175) and *S.epidermidis* (BCI195), the colony forming units (CFUs/mL) adhering to the nanocomposites were assessed after 24 h (Fig. 3A). PLGA/aCaP was used as control material because it does not contain copper. The higher the Cu content of the material, the more pronounced the antibacterial effect (lower CFUs/mL).

Furthermore, SEM images of *S. aureus* adherent on different materials are shown (Fig. 3B). Bacteria were most frequently found within the pores of the meshes. Normally looking *cocci* in process of cell division were found on PLGA. Accumulation of normally looking bacteria in biofilm cluster can be seen on PLGA/aCaP. Deformed bacteria embedded in extracellular matrix in smaller biofilm clusters are visible on PLGA/CuTCP.

### 3.4 CAM assay

**Fig. 4:**
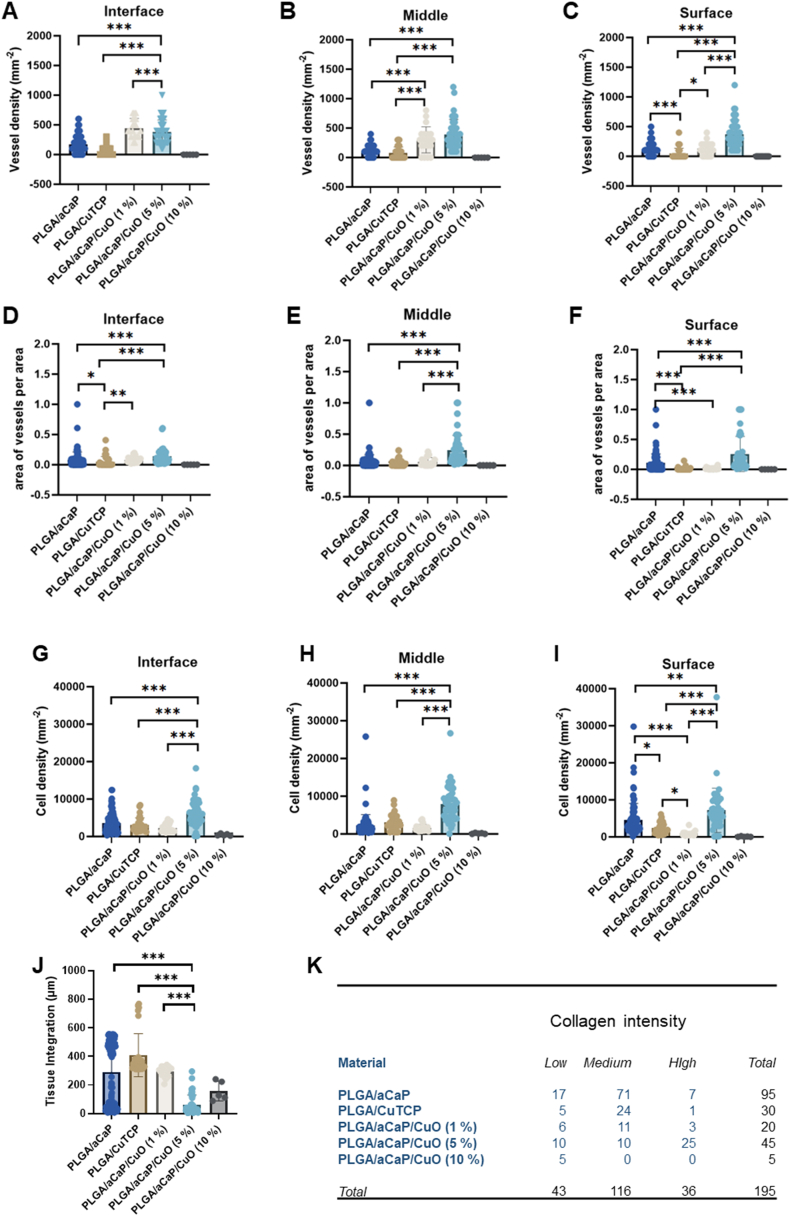
Histological analysis of CAM assay experiments. Vessel density expressed as numbers of vessels per area for the three regions interface, middle and surface (**A-C**, see *Materials and Methods* for definition of regions); vessel area per area (**D-F**); cell density (**G-I**); tissue integration (**J**) and collagen intensity (**K**). As not all data sets were normally distributed, non-parametric *Kruskal-Wallis* test was performed. Significance was considered for p-values <0.05 and indicated as follows: <0.05 (*), <0.01 (**), <0.001 (***). *Note:* Group PLGA/aCaP/CuO (10 %) was not included in the statistical analysis, because data were based on only one egg (all other embryos exposed to this type of nanocomposite died). Pairwise comparison in contingency table revealed p < 0.001 for PLGA/aCaP versus PLGA/aCaP/CuO (5 %) and PLGA/CuTCP versus PLGA/aCaP/CuO (5 %); and p < 0.01 for PLGA/aCaP/CuO (1 %) versus PLGA/aCaP/CuO (5 %). The other pairwise comparisons were not significant. Representative reference images for collagen intensity are given in Supporting Information SI Fig. 1.

As a typical assay for angiogenesis-related questions, the CAM assay was used to determine the vessel density of capillaries growing from the chorioallantoic membrane into the fiber meshes that were gently placed on the CAM for one week. Moreover, area of vessels/area, cell density and tissue integration were quantified; collagen intensity was semi-quantitatively assessed by a contingency table (Figs. 4 and SI Fig. 1).

**Fig. 5:**
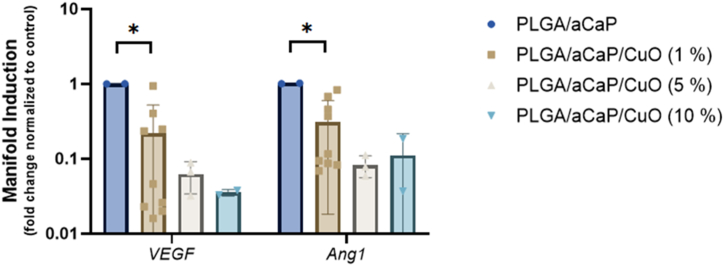
Gene expression of CAM tissue exposed to four different materials. Two typical angiogenic marker genes were analyzed (*VEGF* and *Ang1*). Key: VEGF = vascular endothelial growth factor; Ang1 = angiopoietin 1.

For PLGA/aCaP and PLGA/aCaP/CuO (1, 5 and 10 % of CuO, respectively), qPCR was used to determine the manifold induction of *VEGF* and *Ang1* (two typical pro-angiogenic markers), in the chorioallantoic membrane in contact to these materials for one week (Fig. 5).

The *VEGF* and the *Ang 1* gene expressions of the chorioallantoic membrane were significantly lower when PLGA/aCaP/CuO (1 %) was compared to PLGA/aCaP (0 % of CuO). Among the CuO containing materials, there was no significant difference between each other.

### 3.5 Adipose-derived stem cells seeded on materials

**Fig. 6:**
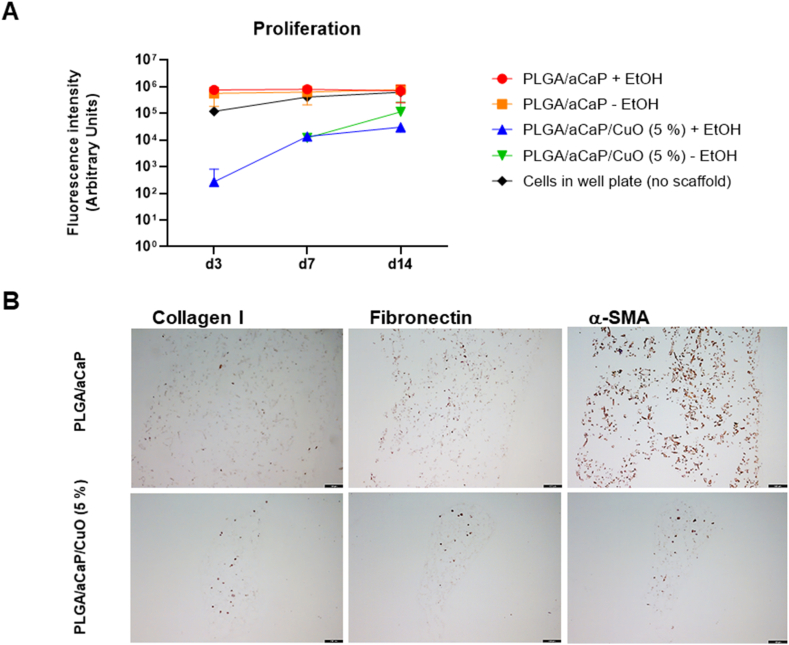
Alamar Blue assay of rabbit ASCs seeded on nanocomposites PLGA/aCaP and PLGA/aCaP/CuO (5 %) that were sterilized with ethanol (+EtOH) or not (-EtOH) in addition to a sterilization with gentamycin/amphotericin B before seeding cells. As a control, cells were cultivated in tissue culture plates referenced as cells in well plate (no scaffold = no nanocomposite) (**A**). Immunohistochemical labeling for collagen I, fibronectin and α-SMA of cell-seeded scaffolds at day 7. Scale bars: 100 μm. (**B**). For negative controls, see the Supporting Information SI Fig. 2. *Key:* d3 = day 3; d7 = day 7 and d14 = day 14, EtOH = ethanol. (For interpretation of the references to colour in this figure legend, the reader is referred to the Web version of this article.)

Because the PLGA/aCaP/CuO (5 %) nanocomposite was the most pro-angiogenic nanocomposite (Fig. 4), rabbit adipose-derived stem cells (ASCs) were seeded onto PLGA/aCaP and PLGA/aCaP/CuO (5 %) and their viability and proliferation was compared, using an Alamar Blue assay (Fig. 6A). Besides sterilization with antibiotics, an additional sterilization step with ethanol (EtOH) was performed for the nanocomposites before cell seeding. While ASCs adhered well on PLGA/aCaP nanocomposites with and without an additional EtOH sterilization, the cells that were seeded with the same initial number adhered less on the PLGA/aCaP/CuO (5 %) nanocomposite, however, they proliferated within the 14-day period of incubation, while cell numbers of ASCs on PLGA/aCaP remained approximately constant over this period.

Immunohistochemical labeling for collagen I, fibronectin and α-SMA at day 14 showed weak but distinguishable brown staining in chromogenic DAB staining for both, PLGA/aCaP as well as PLGA/aCaP/CuO (5 %) (Fig. 6B). For PLGA/aCaP/CuO (5 %), however, the staining intensity was always visibly weaker compared to the control PLGA/aCaP.

## 4 Discussion

The search for an ideal artificial bone substitute in patients with bone loss due to a fracture is ongoing, despite extensive research during the last decades [3,[30], [31], [32]]. As bone biobanks are still scarce, and bone regeneration may turn out challenging, tissue engineered bone is a viable option to fill defects [33]. For the substitution of critical size bone defects, an efficient vascularization has been envisioned by addition of growth factors such as VEGF [34], endothelial precursor cell seeding on the scaffolds [35], a pre-vascularization step [36], microvascular fragments [37] or by arteriovenous loops [38].

While strategies involving cells demand for time consuming and time dependent cell culture, options without living therapeutics allow a production of easy useable off-the-shelf bone substitutes. Among them, transition metals, such as titanium, cobalt and copper, have shown pro-angiogenic properties [18]. Copper may be applied in different forms for bone tissue engineering, and its pro-angiogenic action highly depends on the speciation and chemical (micro) environment of copper. Copper has been shown to modulate angiogenesis by targeting other pro-angiogenic factors, such as HIF1-α, angiogenin, FGF-1 and eNOS [32,[39], [40], [41]]. Moreover, redox active copper is involved in the Fenton reaction [42], leading to the formation of ROS and subsequent VEGF upregulation [43].

It is obvious to apply copper-containing compounds for bone tissue engineering due to their manifold pro-angiogenic effects. Another important fact is the antiviral and antibacterial action of copper compounds, particularly of copper oxide. For example, nanofibrous electrospun meshes were produced with incorporated CuO NPs for anti-viral purposes, resulting in a 70 % reduction of the H1N1 virus after 4 h incubation [44]. Another study supports the antibacterial effect of CuO when incorporated in a chitosan scaffold [45]. Generally, electrospun fibers suitable for BTE [46].

Hence, we aimed at determining the favorite PLGA/aCaP/CuO nanocomposite in a dose-response approach and furthermore aimed at characterizing and comparing the novel PLGA/CuTCP nanocomposite to PLGA/aCaP/CuO, two copper containing electrospun BTE materials with different formulations of the copper incorporation.

The main outcomes of our study with respect to angiogenic effects were the findings of highest vessel and cell densities as well as collagen intensity for PLGA/aCaP/CuO with 5 w/w % CuO NPs in the CAM assay, however, with lowest tissue integration into the electrospun fiber mesh. Moreover, the “hidden” copper in the novel CuTCP NPs resulted in similar angiogenic action like PLGA/aCaP/CuO with 1 w/w % CuO NPs. Finally, the higher the amount of dispersed CuO NPs in PLGA/aCaP/CuO, the more prominent the antibacterial effect against *S. aureus* and *S. epidermidis*. For PLGA/CuTCP and for PLGA/aCaP/CuO with 1 w/w % CuO, similar antibacterial effects were found.

As expected, the characterization of the NPs by XRD revealed low diffraction peaks for the aCaP NPs, but distinct signals for both crystalline copper containing NPs, CuO and CuTCP, respectively (Fig. 1). A prominent peak at 45.8° and a smaller peak at 43.7° were attributed to copper and occurred in XRD spectra of both compounds. In a study by Hegazy et al., the characterization of monoclinic crystal CuO NPs revealed a prominent diffraction peak at 48.8°, which is a bit higher than our signal at 45.8° [47]. Moreover, 38. 6° (prominent) and 35.3° (smaller) diffraction peaks for CuO were reported in a study about transition metal-doped CuO nanopowders [48]. Hence, our copper-related diffraction peaks lie in-between reported values and confirm the presence of copper.

As for the nanocomposites with different amounts of CuO NPs, their random fibers did not vary much in their appearance as assessed by SEM (Fig. 1D). The bioactive divalent copper ion, Cu^2+^, appears upon dissolution of the NPs, (i) situated either on the surface of the PLGA/aCaP/CuO or PLGA/CuTCP fibers; (ii) when NPs are released from the fibers and dissolve in the near proximity of the s nanocomposite or (iii) after uptake by cells.

CuO has a quite low solubility in neutral water (pH = 7.0 at 25 °C), but easily dissolves under acidic conditions [49], according to equation (1).

(1) CuO (s) + 2 H^+^ (aq) → Cu^2+^ (aq) + H_2_O (l)

Studer et al. have measured the Cu^2+^ release from CuO NPs at different pH values (5.5.; 7.0; and 7.4) and confirmed the higher copper release at lower pH values [22]. It was therefore not surprising to find the release kinetics of copper from PLGA/CuTCP in non-complexing buffers [50] to be highly pH dependent as well (Fig. 2 A-D), with ten times more copper at pH = 5.5 than at pH = 7.0 and even twenty times more than at physiological pH = 7.4 after 5 weeks. As lysosomes have a pH = 5.5 inside, release of Cu^2+^ from CuTCP NPs may be high, once these NPs enter the cells and are digested in lysosomes. However, in the extracellular space, only small amounts of copper ions can be expected, caused by the higher pH and low solubility. Indeed, the solubility of the CuTCP NPs strongly depends on the compartment, very similar to the reported findings for CuO NPs [22].

The amount of released copper depended on the concentration of CuO NPs dispersed in the PLGA/aCaP/CuO fibers (Fig. 2 E). As expected, under otherwise same conditions, more copper was released from the nanocomposite with 10 w/w % CuO than from nanocomposites with lower CuO concentrations. However, the differential increase of released copper from 1 w/w % CuO to 5 w/w % CuO was much more pronounced (factor 74) than from 5 w/w % CuO to 10 w/w % CuO, where it was a factor of 2, which would make sense as the double amount of CuO NPs was used in the 10 % compared to the 5 % version. We speculate that the CuO NPs in the 1 w/w % formulation were partly associated with the 39 w/w % aCaP NPs and mostly buried within the PLGA/aCaP/CuO fibers, so that the release of copper was retarded. In contrast, with 5 or 10 w/w % CuO and correspondingly less aCaP NPs, many more CuO NPs were on the fiber surface exposed to the release medium compared with the 1 w/w % formulation. Furthermore, they were to a lower part associated with the aCaP NPs.

Bacterial infection of bone or bone marrow adjacent to or on implants is a serious post-surgical complication, caused by a nosocomial contamination [51]. Such infections are often associated with avascular necrosis of bone, and antibiotic therapy in addition to surgical debridement is needed [52]. A major pathogen during bone infections has been reported to be *Staphylococcus aureus* [53]. To counteract these complications, novel implant materials have been developed that contain an antibacterial component. For example, silver has been applied to prevent prosthetic joint infections [54], or silver-containing nanocomposites have been shown to act more efficient and over longer periods against *E. coli* than tetracycline soaking did [8]. Other examples cover magnesium oxide in hydroxyapatite that showed to be antibacterial in experiments with *Pseudomonas aeruginosa* and *Staphylococcus epidermidis* [55].

Our experiments with clinical isolates of *S. aureus* (BCI 175) and *S. epidermidis* (BCI 195) clearly showed an antibacterial effect with increasing copper content of the materials. All pairwise comparisons between the 5 materials showed significant differences, except for one pair, the PLGA/aCaP/CuO (1 w/w %) and the PLGA/CuTCP nanocomposites, that exhibited very similar antibacterial activity (Fig. 3). This result can be expected because the Cu fraction is approximately the same for both nanocomposites, based on the following calculation. The novel PLGA/CuTCP had 2 % of Cu within the CuTCP NPs which themselves had a 40 w/w % fraction in the nanocomposite, resulting in 0.02 x 0.40 = 0.008 fraction; very similar to the 1 w/w % of the CuO NPs within the PLGA/aCaP/CuO composite, which results in (63.546/79.545) x 1 % = 0.008 fraction, where 63.546 refers to the M_w_(Cu) and 79.545 is the M_w_(CuO), respectively.

The main subject of interest was to assess the angiogenic response evoked by the different nanocomposites, using the CAM assay, (Fig. 4). While the material with 5 % CuO NPs exhibited the highest vessel density and vessel area/area in all of the three regions (interface, middle, surface), going along with significantly higher cell densities, the tissue integration into the material was lowest for this material, significantly lower than all other groups (Fig. 4). These results suggest an anti-proliferative effect of CuO NPs, inhibiting tissue ingrowth to a certain degree, while on the other hand supporting angiogenic properties.

The copper speciation plays a fundamental role for angiogenesis. While CuO NPs have been reported to be toxic depending on their concentration [56] and may therefore lead to an anti-angiogenic effect, released copper ions, such as the divalent Cu^2+^ and after reduction the monovalent Cu^+^, are involved in redox reactions that lead to the formation of ROS [43]. At low levels, such species promote angiogenesis by induction of VEGF expression [57]. By comparing different amounts of CuO NPs incorporated in the nanocomposite materials here, we found highest angiogenic induction in the CAM assay for a 5 % CuO content (Fig. 4). At 10 % CuO NPs, we found severe toxic effects, which was pronounced by the high fraction of dead chicken embryos in the CAM assay, resulting in only one survivor out of 10, explaining the unequal sample size. In contrast, at low concentrations like 1 % CuO NPs or in the Trojan horse formulation of novel CuTCP NPs, pro-angiogenic effects were only observed by trend when compared to the control.

These histological results from the CAM assay were, however, not confirmed for *VEGF* and *Ang1* gene expressions of chorioallantoic membrane tissue harvested from underneath the nanocomposites (Fig. 5). As one of the central growth factors in angiogenesis, *VEGF* induction promotes *de novo* vessel formation [58]. Also *Ang1* acts pro-angiogenic, while *Ang2* is a vascular disruptive agent with antagonistic activity through the Tie2 receptor [59], the same receptor targeted by the ligand *Ang1* [60]. While *Ang1* leads to a stabilization of (newly formed) vessels, *Ang2* inhibits this stabilization – only in combination with *VEGF* does *Ang2* also induce angiogenesis, indicating the complexity and interplay of these regulatory factors. Our findings of downregulated *VEGF* and *Ang1* gene expression through the presence of CuO NPs within the nanocomposites does not reflect the pro-angiogenic effects observed in the histological readouts of the *in ovo* CAM assay (Fig. 4), but may be explained by gene expression dynamics, where upregulation of those pro-angiogenic factors may have been already over at the endpoint of 7 days in the CAM assay, as a consequence of transcriptional dynamics [61].

Finally, cell-material interactions were assessed by seeding rabbit adipose-derived stem cells (ASCs) on the nanocomposites (Fig. 6A). The presence of CuO NPs inhibits to a certain extent the initial adherence of the ASCs on the fiber meshes; however, over a period of 14 days, the cells proliferate well. In contrast, the control material PLGA/aCaP showed a good initial ASC adherence on the fibers, but over time, no substantial increase in proliferation had been detected. Such behavior is congruent with the reduced tissue integration for the 5 % formulation observed in the CAM assay (Fig. 4J). Moreover, cells seeded on these artificial bone constructs expressed ECM markers, such as collagen I, fibronectin and α-SMA, which we delineated by immunohistochemical labellings of cell-seeded nanocomposites (Fig. 6B). After a 2-week incubation, intensities were, however, only weak in general and weaker in the CuO containing PLGA/aCaP/CuO compared to the control, indicating a reduced ECM formation of ASCs seeded on the 5 w/w % CuO containing material. We conclude that ASCs are highly sensitive towards the CuO NPs incorporated in the nanocomposite. It has been reported that after a 5-day exposition to 25 μm CuSO_4_, cellular respiration of human ASCs was decreased as well as gene expression of *collagen I* [62], supporting our determination of lower ECM formation in the 5 w/w % CuO containing material compared to the control.

## 5 Limitations

As with every work, limitations have to be considered. 1) Longer incubation periods of cell-seeded nanocomposites may have clarified IHC labellings and enhance intensities. 2) The PLGA/aCaP/CuO nanocomposite with 10 w/w % CuO was difficult to examine, with only 1 egg out of 10 that survived. Hence, concentrations lying between 5 and 10 w/w % of CuO should be further explored. 3) *In vivo* experiments where bone defects are substituted by these artificial bone void fillers are still missing.

## 6 Conclusion

In sum, we have characterized the three kinds of NPs (aCaP, CuO and novel CuTCP). All novel nanocomposites showed promising characteristics with respect to pro-angiogenic features, particularly the PLGA/aCaP/CuO (5 w/w %) that was elicited as the favorite in the dose-response approach. In addition, prominently expressed antibacterial properties have been verified against clinical isolates *S. aureus* and *S. epidermidis*, where the nanocomposites exhibited higher antibacterial characteristics with increased CuO NP content. As for the newly synthesized special NPs based on copper-doped tricalcium phosphate, they acted comparable to the PLGA/aCaP/CuO (1 w/w %) when applied as PLGA/CuTCP, both with respect to pro-angiogenic and antibacterial activity, respectively.

We conclude that (i) PLGA/aCaP/CuO (1 w/w %) and PLGA/CuTCP have very similar biological activity concerning angiogenesis and antibacterial qualities and can be used interchangeably for BTE; and (ii) PLGA/aCaP/CuO (5 w/w %) is the favorite bone substitute compared to lower (1 %) or higher (10 %) CuO fractions within the nanocomposite. However, *in vivo* experiments in animal models have still to be performed to pave the way for their future clinical application as bone void fillers for critical size bone defects.

## Funding

This research was supported by the Hartmann Müller Foundation Zurich (“Bone tissue engineering”) and the 10.13039/100000001Swiss National Science Foundation *SNSF* project number 310030_197578.

## Ethics

The CAM assay does not need any license from the veterinary office until ID 14 according to Swiss legislation (TSchV, Art. 112).

## Data availability

Upon request, data are provided.

## CRediT authorship contribution statement

**Lukas Näf:** Writing – review & editing, Writing – original draft, Methodology, Investigation, Formal analysis, Data curation. **Iris Miescher:** Writing – review & editing, Methodology, Investigation, Formal analysis. **Lara Pfuderer:** Writing – review & editing, Methodology, Investigation, Formal analysis, Data curation. **Tiziano A. Schweizer:** Writing – review & editing, Writing – original draft, Methodology, Investigation, Data curation. **David Brunner:** Investigation, Formal analysis. **Johannes Dürig:** Investigation, Formal analysis. **Olivier Gröninger:** Writing – review & editing, Methodology, Data curation. **Julia Rieber:** Investigation. **Gabriella Meier-Bürgisser:** Writing – review & editing, Methodology. **Katharina Spanaus:** Writing – review & editing, Methodology, Formal analysis, Data curation. **Maurizio Calcagni:** Writing – review & editing, Supervision. **Philipp P. Bosshard:** Writing – review & editing, Resources. **Yvonne Achermann:** Writing – review & editing, Resources. **Wendelin J. Stark:** Writing – review & editing, Supervision, Resources, Conceptualization. **Johanna Buschmann:** Writing – review & editing, Writing – original draft, Validation, Supervision, Resources, Project administration, Funding acquisition, Conceptualization.

## Declaration of competing interest

The authors declare the following financial interests/personal relationships which may be considered as potential competing interests:Johanna Buschmann reports financial support was provided by 10.13039/501100009396University Hospital Zurich. Johanna Buschmann reports a relationship with University Hospital Zurich that includes: employment. If there are other authors, they declare that they have no known competing financial interests or personal relationships that could have appeared to influence the work reported in this paper.
