# Supplementary material for: Pro-angiogenic and antibacterial copper containing nanoparticles in PLGA/amorphous calcium phosphate bone nanocomposites
Source: Heliyon. 2024 Mar 4;10(5):e27267. doi: 10.1016/j.heliyon.2024.e27267 (PMC10937708; doi:10.1016/j.heliyon.2024.e27267)
Supplement: Multimedia component 1 [file mmc1.docx]

**Supporting Information**

**Pro-angiogenic and antibacterial copper containing nanoparticles in PLGA/amorphous calcium phosphate bone nanocomposites**

Lukas Näf^a^, Iris Miescher^a^, Lara Pfuderer^b^, Tiziano A. Schweizer^c^, David Brunner^a^, Johannes Dürig^a^, Olivier Gröninger^b^, Julia Rieber^a^, Gabriella Meier-Bürgisser^a^, Katharina Spanaus^d^, Maurizio Calcagni^a^, Philipp P. Bosshard^c^, Yvonne Achermann^c^, Wendelin J. Stark^b^, and Johanna Buschmann^a ^[[1]](#footnote-1)^*^

^a^ Department of Plastic Surgery and Hand Surgery, University Hospital of Zürich,

Rämistrasse 100,8091 Zürich, Switzerland

^b^ Institute for Chemical and Bioengineering, Department of Chemistry and Applied Biosciences, ETH Zurich, CH-8093 Zurich, Switzerland

^c^ Department of Dermatology, University Hospital Zurich, University of Zurich, Rämistrasse 100, 8091 Zurich, Switzerland

^d^ Clinical Chemistry, University Hospital Zurich, 8001 Zurich, Switzerland


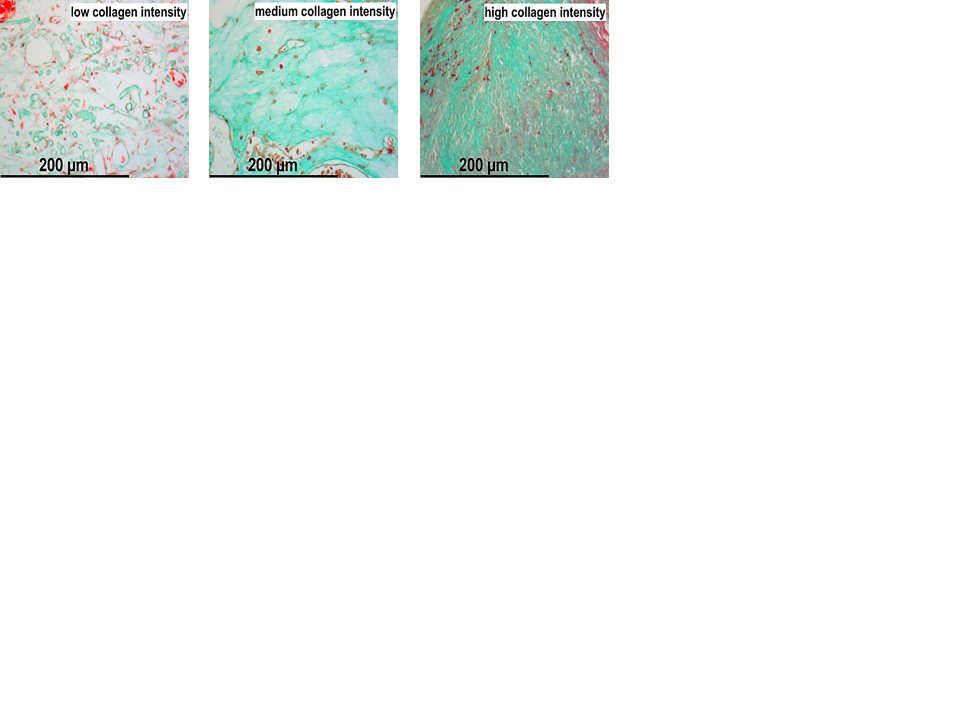


**SI Figure 1** Representative images for low, medium and high collagen intensity as assessed in the CAM assay after Masson Goldner Trichrome staining. Collagen can be seen as greenish to turquois colored tissue. These images were used as reference images for the semi-quantitative analysis with data presented in Figure 4J as contingency table.


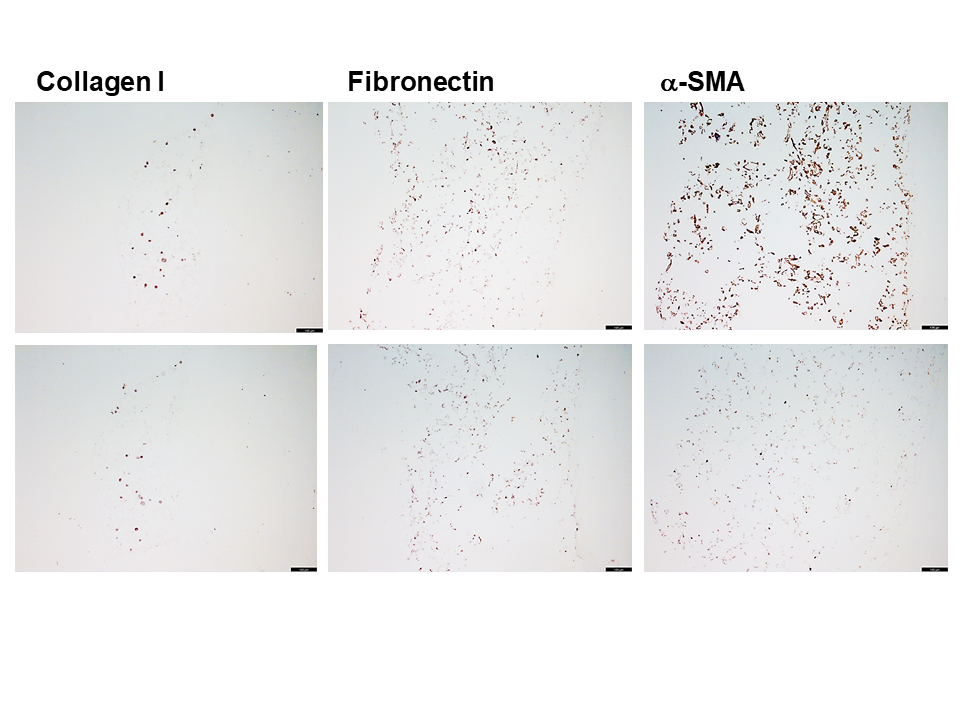


**SI Figure 2** Representative images for immunohistochemical labeling of collagen I, fibronectin and α‑SMA (upper row) with corresponding negative controls (lower row) in 3 μm thick sections of ASCs seeded on PLGA/aCaP nanocomposites. Scale bar: 100 µm.

1. * Corresponding Author

   E-mail address: Johanna.Buschmann@usz.ch [↑](#footnote-ref-1)
